# Palmitate Inhibits SIRT1-Dependent BMAL1/CLOCK Interaction and Disrupts Circadian Gene Oscillations in Hepatocytes

**DOI:** 10.1371/journal.pone.0130047

**Published:** 2015-06-15

**Authors:** Xin Tong, Deqiang Zhang, Blake Arthurs, Pei Li, Leigh Durudogan, Neil Gupta, Lei Yin

**Affiliations:** Department of Molecular & Integrative Physiology, University of Michigan Medical School, Ann Arbor, Michigan, United States of America; McGill University, CANADA

## Abstract

Elevated levels of serum saturated fatty acid palmitate have been shown to promote insulin resistance, increase cellular ROS production, and trigger cell apoptosis in hepatocytes during the development of obesity. However, it remains unclear whether palmitate directly impacts the circadian clock in hepatocytes, which coordinates nutritional inputs and hormonal signaling with downstream metabolic outputs. Here we presented evidence that the molecular clock is a novel target of palmitate in hepatocytes. Palmitate exposure at low dose inhibits the molecular clock activity and suppresses the cyclic expression of circadian targets including *Dbp*, *Nr1d1* and *Per2* in hepatocytes. Palmitate treatment does not seem to alter localization or reduce protein expression of BMAL1 and CLOCK, the two core components of the molecular clock in hepatocytes. Instead, palmitate destabilizes the protein-protein interaction between BMAL1-CLOCK in a dose and time-dependent manner. Furthermore, we showed that SIRT1 activators could reverse the inhibitory action of palmitate on BMAL1-CLOCK interaction and the clock gene expression, whereas inhibitors of NAD synthesis mimic the palmitate effects on the clock function. In summary, our findings demonstrated that palmitate inhibits the clock function by suppressing SIRT1 function in hepatocytes.

## Introduction

Obesity and its associated metabolic complications have become epidemic due to the sedentary lifestyle and consumption of high-sugar and high-fat foods. Obesity greatly increases the risk of diabetes by lowering insulin sensitivity and promoting chronic low-grade inflammation in the liver and adipose tissues [1, 2]. In animal models of high-fat diet-induced obesity, elevated levels of saturated free fatty acids (FFA) in circulation have been considered a primary factor that promotes insulin resistance in key metabolic tissues such as liver, skeletal muscles and pancreatic β-cells [3–5]. Several cellular targets including JNK [6], IKKβ [7], ER stress [8], ceramide [9, 10], as well as oxidative stress [11] have been identified to link FFA to insulin resistance in hepatocytes. Interestingly, palmitate, one of major FFA, was found to influence the molecular clock function in an immortalized hypothalamic cell line and alter the expression of the neuropeptide NPY [12, 13]. Given its potent metabolic effects on hepatocytes, it is of great interest to study whether palmitate directly modulates the molecular clock function in hepatocytes.

In recent years, circadian rhythms have emerged as a new regulator of metabolic homeostasis [14, 15]. Mouse models with either deletion or mutation of the core clock gene such as *Bmal1 [16–19], Clock* [18, 20], *Per2* [21], *ROR-α [22, 23]*, and *Nr1d1* [24, 25] have demonstrated various metabolic phenotypes, indicating an essential role of clock genes in metabolic regulation. Reciprocally, metabolic events can impact clock activity and function [26, 27]. Timing of food intake, such as restrictive feeding can alter the expression pattern of key clock genes in the liver [28, 29]. High fat content in food also has been shown to influence the clock oscillation and function in various high-fat diet (HFD)-treated animal studies [30–32]. Kohsaka et al demonstrated that 6-week HFD altered the locomoter activity, clock genes, and nuclear receptors in various tissues of C57BL/6 male mice [31]. Hsieh et al showed that 11-month HFD also disrupted clock gene oscillations in the liver and kidney of C57BL/6 male mice [30]. However, Yanagihara et al reported no effect of HFD on the circadian clock in C57BL/6 female mice [32]. In a recent study, HFD feeding was shown to reprogram circadian gene oscillations by inducing cyclic activation of transcription regulators that have not been directly associated with the circadian clock [33]. Overall, the effects of HFD on circadian clock in animal studies seem to be gender-, duration-, and pathway-specific. So far, the signaling pathways directly connecting nutritional status and cellular clock activity remain largely unknown.

At the molecular level, the circadian rhythm is generated through an intertwined transcription and translational feedback loop system consisting of a positive limb made of transcription activators (BMAL1, CLOCK) and a negative limb that includes repressors (PER, CRY, and REV-ERB*α*) [34–36]. The oscillating activities of those positive and negative loops drives rhythmic expression of the metabolic output genes through clock protein-dependent transcriptional events. Both BMAL1 and CLOCK are bHLH (basic helix-loop-helix) PAS (Per-Arn-Sim)-domain containing proteins. The protein complex of BMAL1-CLOCK is necessary for transactivation of numerous circadian targets. However, how BMAL1-CLOCK interaction and their transcriptional activity are controlled during various stress conditions is unclear. Several metabolic regulators have been found to participate in regulating BMAL1-CLOCK transcriptional activity, in particular the NAD-dependent deacetylase SIRT1 [37–39]. Loss of the molecular clock activity and delayed PER2 protein degradation were observed in *Sirt1* ^*-/-*^ mouse embryonic fibroblast [40]. It was also reported that SIRT1 interacts with the BMAL1-CLOCK complex, deacetylates BMAL1, and suppresses its transcriptional activities [41]. Pharmacological manipulation of SIRT1 activity was also shown to affect the molecular clock activity in mouse embryonic fibroblast [42]. Because SIRT1 acts as an intracellular metabolic sensor [43] and its expression and activity vary dependent on the cell type [44], it is plausible that SIRT1 directly couples intracellular energy status and the molecular clock activity in a cell-type specific manner.

In our current study, we presented evidence that palmitate directly targets the molecular clock in hepatocytes. Exposure to low-dose palmitate suppresses the circadian oscillations of clock genes. Palmitate treatment causes destabilization of BMAL1-CLOCK interaction. SIRT1 activator restores BMAL1-CLOCK interaction and clock gene expression in palmitate-treated hepatocytes. Our results suggest that palmitate might mediate the HFD-induced suppression of the molecular clock in the liver via the SIRT1 pathway.

## Materials and Methods

### Generation of recombinant adenoviruses and reagents

Adenoviruses were generated using pAdViraPower system (*Life Technologies*) for Ad-Clock, pAdEasy system (*Agilent*) for Ad-Bmal1. Over-expression of BMAL1 or CLOCK in primary hepatocytes was achieved by transducing primary hepatocytes with the concentrated adenovirus at 1x10^9^ pfu/mL. SIRT1 inhibitor EX527 was purchased from *Selleckchem*. Resveratrol and CAY10591 were from *Cayman*. FK866 was from *Sigma*.

### Synchronization of cultured hepatocytes

Mouse hepatoma Hepa1c1c-7 (Hepa1) cells were used in the synchronization study [45]. Confluent cells were serum-shocked (50% horse serum) for 2 hr and then treated with either fatty acid-free bovine serum albumin (BSA) or palmitate (50 μM) in 0.5% BSA. Cells then were collected for both mRNA and protein analysis at 4-hr intervals between 24 hr and 48 hr time points.

### Isolation of primary mouse hepatocytes

Primary mouse hepatocytes were isolated from 8–10 wk old wild type C57BL6 mice using a two-step collagenase digestion with 100 U/mL collagenase in HBSS at pH 7.4. After dissection, the liver was placed in DMEM and carefully pulled apart to release hepatocytes. Hepatocytes in DMEM were passed through a 100 μM cell strainer and then spun at 50 X g for 1 min. The pellet was resuspended in DMEM and then spun at 50 X g for 10 min in a Percoll gradient to remove dead hepatocytes. After washing with DMEM at 50 X g for 10 min and checking by Trypan Blue staining, hepatocytes were cultured on collagen-coated plates. All animal experiments were approved by the Institutional Animal Care and Research Advisory Committee at the University of Michigan Medical School.

### Protein preparation and western blot

Cell pellets were lysed in ice-cold RIPA (50 mM Tris, pH 8.0, 150 mM NaCl, 2 mM EDTA, 0.5% NP-40, 0.1% SDS, 0.5% sodium deoxycholate) buffer supplemented with 1x protease inhibitor and 50 mM NaF and incubated on ice for 20 min. Protein lysates were cleared by centrifugation at 14,000 rpm at 4°C for 10 min. Supernatants were collected and quantified using BioRad protein assay kit. For isolation of clock proteins in nuclear fractions, cells or tissues were first exposed to hypotonic buffer (10 mM Tris-HCl, pH 8.0, 10 mM NaCl, 3 mM MgCl_2_, 0.2% NP-40, and 300 mM Sucrose) and cytosolic fractions were separated by low-speed centrifugation. Nuclear pellets were then re-suspended in RIPA buffer and centrifuged at 14,000 rpm at 4°C to obtain nuclear fractions. Blots were probed with the following primary antibodies: BMAL1, CLOCK AKT, p38, SIRT1, p53 (Santa Cruz Biotechnology); β-tubulin (Sigma); AKT-phospho (Cell Signaling, AKT phosphorylation at Ser473), p38-phospho (Cell Signaling, p38 dual phosphorylation at Thr180/Tyr182), and p53-Acetyl (Cell signaling, Lys382)

### Immunoprecipitation

The standard immunoprecipitation method was described previously (65). For detecting the protein interaction between BMAL1 and CLOCK, cells were harvested after hr treatment with either BSA or palmitate following 2 hr of serum shock. Cell lysates were then incubated with specific antibodies (rabbit IgG control or anti-BMAL1) overnight at 4°C. The immune-complexes were captured by adding 30 μL of Protein A-Sepharose beads and incubating at 4°C for 1 hr. Beads were washed 5 times in RIPA buffer and eluted in 30 μl 2X SDS loading buffer. Western blotting was performed to detect the presence of targeted proteins with specific antibodies. For affinity purification, cells were lysed in lysis buffer and 1 mg of protein lysate incubated with 20 μL of Streptavidin magnetic beads (*GE Heathcare*) for 6 hr. The beads were then washed with lysis buffer for 5 times and eluted in 20 μL of 2X SDS loading buffer.

### RNA extraction and RT-qPCR

Total cellular RNA extraction was performed with TRIzol reagent (Invitrogen, Carlsbad, CA) and chloroform. cDNA was synthesized using Verso cDNA kit (Thermo Fisher Scientific, Surrey, UK) and subjected to qPCR using Absolute Blue SYBR Green ROX Mix (Thermo Fisher Scientific, Surrey, UK) on an ABI 7900 HT thermal cycler (Applied Biosystems, Foster City, CA). The value of each cDNA was calculated using the ΔΔCt method and normalized to the value of housekeeping gene control (18 S RNA). Data were plotted as fold change. The primer sequences for mouse clock and control genes were reported previously [46]. The primers for other genes for this study are listed in Table 1. These qPCR primers were designed to span exon-exon junctions by using the NCBI on-line primer design software. The selected primer shows no homology to other sequences in the mouse genome at least at last 3 nucleotides at 3’ end. The amplification efficiency of primer set were monitored by delta CT method to make sure at least 2 cycle difference in a series of diluted samples. The PCR products were verified to be the right size by gel electrophoresis.

**Table 1:** QPCR primers that were used in this study.

Name	Forward	Reverse
*Bmal1*	GCAGTGCCACTGACTACCAAGA	TCCTGGACATTGCATTGC
*Clock*	CATCAACTCAGCAGAGTCAACA	AGGCTGGGAAATCACCATCG
*Cry1*	CGCAGGTGTCGGTTATGAGC	ATAGACGCAGCGGATGGTGTCG
*Cry2*	ATCATTGGCGTGGACTACCC	ATCATTGGCGTGGACTACCC
*Nr1d1*	CCCTGGACTCCAATAACAACACA	GCCATTGGAGCTGTCACTGTAC
*Per2*	CAGCTGCCCTCCCGGGATCT	TCCTGGACATTGCATTGC
*Dbp*	GGAACTGAAGCCTCAACCAAT	CTCCGGCTCCAGTACTTCTCA
*Pgc-1α*	TGGACGGAAGCAATTTTTCA	TTACCTGCGCAAGCTTCTCT

### Transfection and luciferase assay

Cells were plated in a 24-well plate overnight before Lipofectamine 2000 (Invitrogen)-mediated transfection with the *mPer2* promoter-driven luciferase reporter alongside expression vectors for BMAL1 and CLOCK. 24 hr post transfection, cells were synchronized with 50% horse serum for 2 hr and switched back to serum-free medium supplemented with either BSA or palmitate. 24 hr later, cells were lysed for luciferase activity assay measurement on a BioTek Synergy 2 microplate reader. β–galactosidase construct was also co-transfected in each well for normalizing luciferase activity.

### Statistical analysis

Student’s *t*-test was used to determine the significance of difference between two groups with *p* value < 0.05. One-way ANOVA (Prism software) along with *post-hoc Tukey’s* test was used to test the difference between more than two groups. For cosinor analysis of oscillations of circadian genes, JTK software was used to generate values of amplitude and *p*-values [47].

## Results

### Palmitate inhibits the molecular clock function in mouse hepatocytes

Several studies have already shown high-fat diet (HFD) feeding impairs circadian rhythmicity [48, 49] and dampens chromatin recruitment of BMAL1-CLOCK transcriptional complex [33]. We also observed that an 8-wk-HFD is sufficient to dampen diurnal oscillations of core clock genes in the liver and the spleen (data not shown). However, it remains unclear about whether disruption in these peripheral clocks is a primary response to HFD or secondary to obesity and insulin resistance following HFD feeding. It has been well documented that HFD causes elevated levels of serum free saturated fatty acids [50]. Among these fatty acids, the long-chain (C16:0) fatty acid palmitate is particularly detrimental to hepatocytes by inducing lipotoxicity such as insulin resistance, oxidative stress, and cell death at high dose and for prolonged treatment [11, 51, 52]. However, its direct effects on the molecular clock in hepatocytes have not been examined.

**Fig 1:**
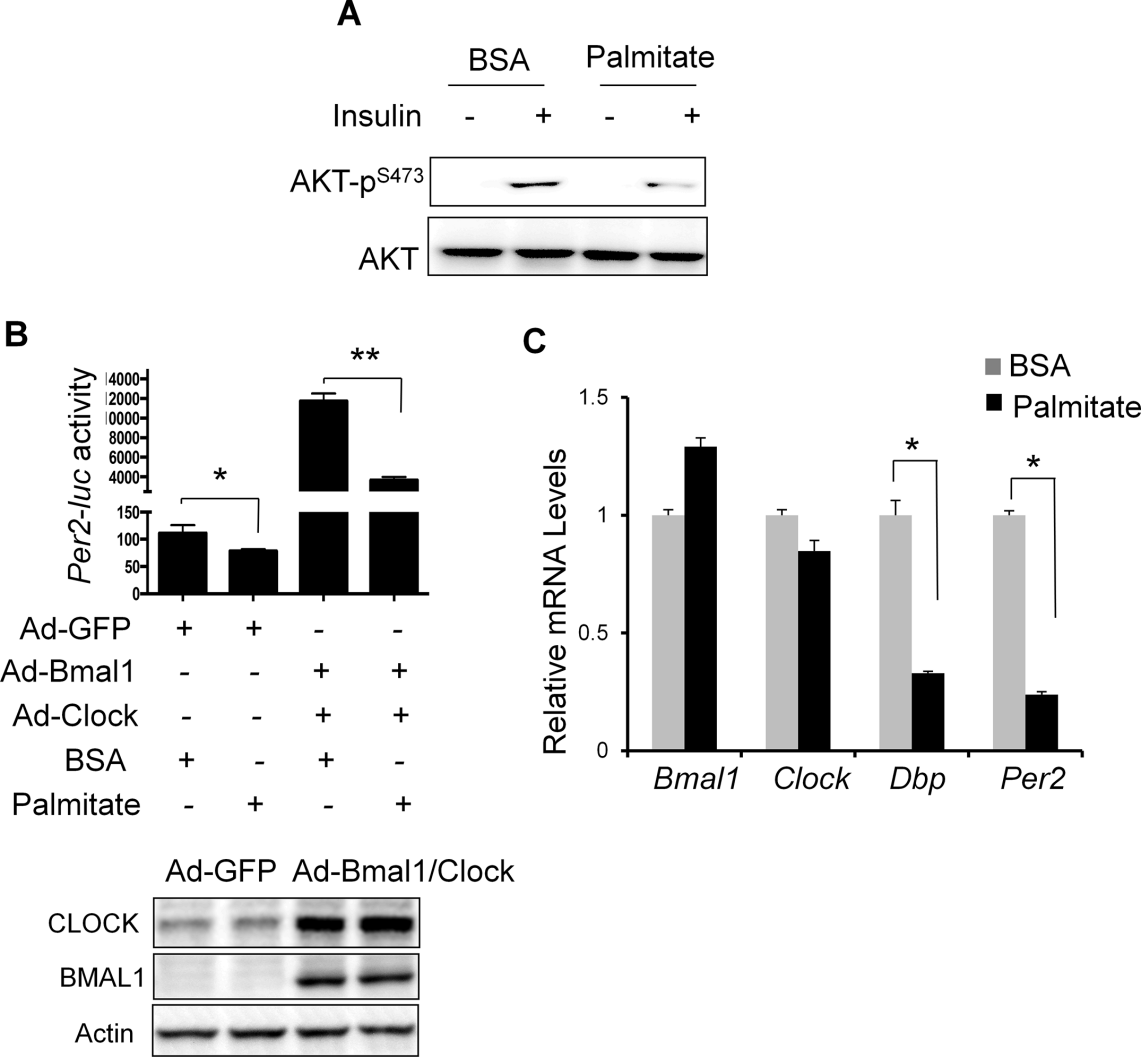
Saturated fatty acid palmitate suppresses the molecular clock activity in mouse hepatocytes. **(A)** Palmitate inhibits insulin-stimulated AKT-P in primary mouse hepatocytes (PMHs). PMHs were exposed to palmitate (50 μM) for 16 hr before stimulation with insulin (10 nM) for 30 min. The levels of cellular AKT and AKT-p^S473^ (Ser 473) were examined by immunoblotting. **(B)** Palmitate represses the induction of *Per2-luc* activity by BMAL1 and CLOCK. Hepa1 cells was transfected with *Per2-luc* before transduction with either Ad-GFP control or Ad-Bmal1-Flag plus Ad-Clock. 24 hr later, cells were treated with palmitate at 50 μM or BSA (vehicle control) for additional 16 hr before luciferase assay. Luciferase activity was normalized to β–gal activity. Data were plotted as mean + SD (n = 3). The protein levels of BMAL1 and CLOCK following adenoviral transduction were examined by immunoblotting (bottom panel). **(C)** Palmitate reduces circadian gene expression in PMHs. PMHs were treated with either BSA (vehicle control) or palmitate at 50 μM for 24 hr. Cells were then harvested for RT-qPCR. The results were plotted as fold change using the value of BSA-treated samples as 1. Data were plotted as mean + SD (n = 4). *p < 0.05 and ** *p* < 0.01.

We first determined the appropriate concentration of palmitate for our *in vitro* circadian study because high-dose of palmitate (over 400 μM) triggers cell apoptosis [53]. As insulin resistance is one of the hallmarks of palmitate-induced lipotoxicity, we tested a wide range of palmitate dosages for its effects on insulin-induced AKT phosphorylation (AKT-P at Ser473) (data not shown). In freshly isolated primary mouse hepatocytes (PMHs), we observed that treatment of palmitate at as low as 50 μM overnight is sufficient to reduce AKT-p^S473^ without decreasing the total AKT expression compared with BSA (**Fig 1A**). In addition, palmitate treatment at this concentration does not cause a significant decrease in cell viability, determined by dimethylthiazolyl diphenyltetrazolium bromide (MTT) assay (data not shown).

To test whether low dose of palmitate (50 ~ 100 μM) affects the molecular clock function in hepatocytes, we examined the effect of palmitate treatment on the activity of a mouse *Per2* promoter-driven luciferase reporter (*Per2-luc*) in a mouse hepatoma Hepa1c1c-7 (Hepa1) cell line. *Per2-luc* shows a low basal activity but is potently induced by adenovirus-mediated overexpression of both BMAL1 and CLOCK, the key transcription complex of the molecular clock in hepatocytes **(Fig 1B)**. However, addition of palmitate significantly reduces *Per2-luc* in the presence or absence of *Bmal1* and *Clock* over-expression. Consistent with the *Per2-luc* reporter assay, palmitate treatment for 24 hr significantly reduces the mRNA levels of two classical circadian genes *Dbp* and *Per2* in PMHs **(Fig 1C)**. However, the expression of *Bmal*1 and *Clock* is not affected by palmitate. Thus, we showed evidence that low dose palmitate treatment can suppress the *Per2-luc* activity induced by the BMAL1-CLOCK complex and reduce the expression of a subset clock output genes in mouse hepatocytes.

### Palmitate represses the clock gene oscillations in cultured hepatocytes

**Fig 2:**
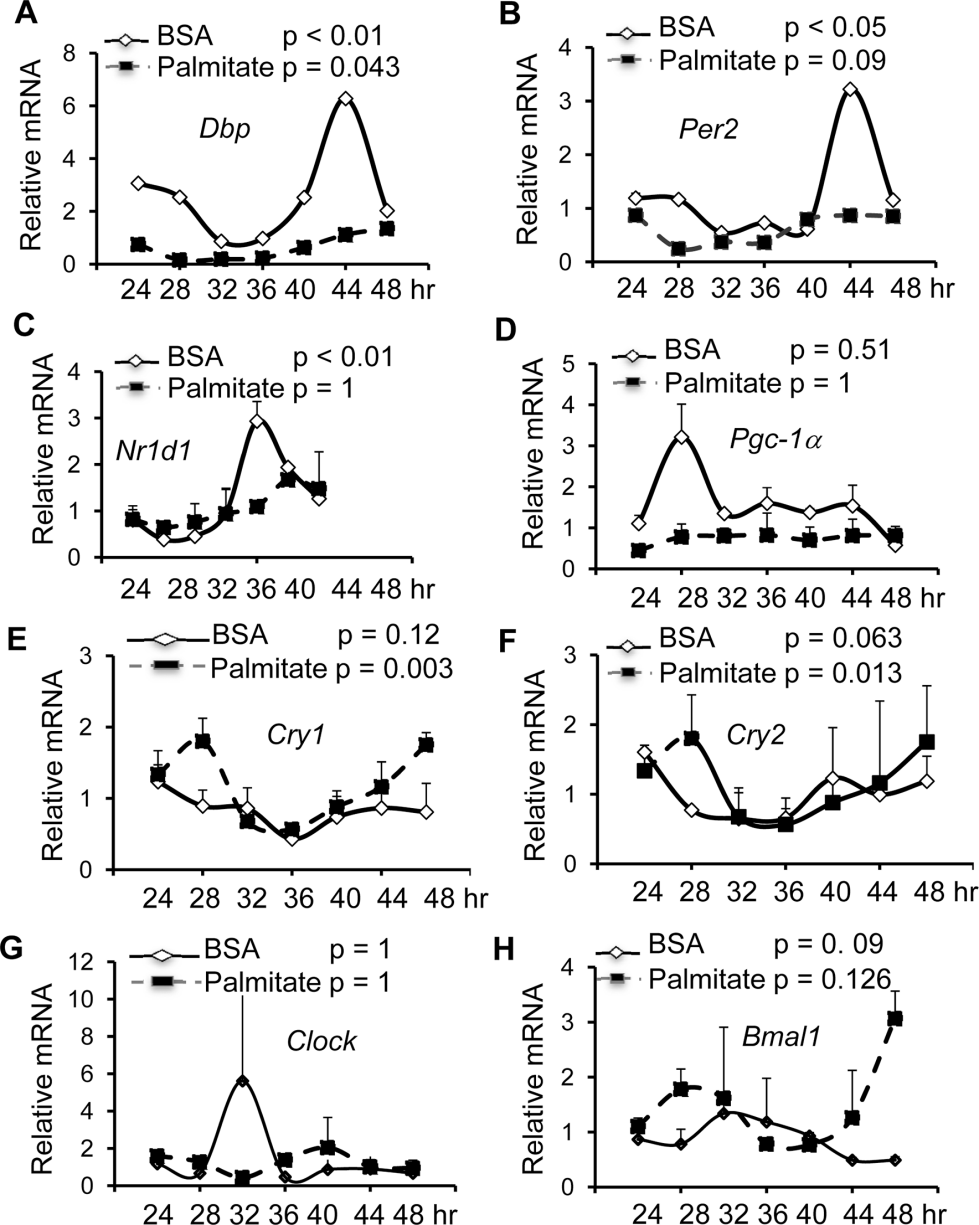
Palmitate suppresses circadian oscillations of clock genes in cultured hepatocytes. Hepa1 cells were synchronized by 2-hr serum shock before palmitate treatment. Cell RNA samples were then harvested at 4 hr intervals in the following 24 hr period for RT-qPCR analysis. Three clock genes including *Dbp* **(A)**, *Per2* **(B),** *Nr1d1* **(C)**, *Pgc-1*α **(D)**, Cry1 **(E)**, Cry2 **(F)**, *Bmal1* **(G)**, and *Clock* **(H)** were examined and plotted as fold change (mean + range, n = 2). The JTK software was used to perform cosinor analysis on the data sets. All *p*-values were shown in Fig (2A-H) and the amplitude values were shown in Table 2.

PMHs, once isolated, maintain their hepatocyte morphology for only a short period of time, making them a less ideal model for studying circadian oscillations in a longer duration. To determine the effect of palmitate on the oscillations of key clock genes, we turned to Hepa1 cells, a previously reported *in vitro* hepatocyte model for studying the molecular clock in our lab [45]. After serum shock, Hepa1 cells were exposed to either BSA or palmitate for 48 hr. The mRNA oscillations of *Bmal1*, *Clock*, *Cry1*, *Cry2*, *Dbp*, *Per2*, *Nr1d1*, *and Pgc-1α* were measured between 24 hr and 48 hr post serum shock. During the entire circadian cycle, *Dbp*, *Per2*, *and Nr1d1* mRNA levels show robust oscillations with about 5 to 8-fold difference between CT (Circadian Time) 32 and CT 44 in the BSA control cells. However, in the presence of palmitate, oscillations of clock genes including *Dbp*, *Nr1d1 and Per2* are severely dampened (**Fig 2A–2C & Table 2**). Palmitate treatment also reduces oscillations of *Pgc-1α*, a key metabolic regulator known to be a target of the molecular clock [54] (**Fig 2D)**. Meanwhile, palmitate does not seem to dampen the cyclic expression of *Bmal1*, *Cry1 and Cry2* (**Fig 2E and 2F**). Taken together, our results demonstrated a potent but selective inhibition of oscillations of a panel of clock genes and clock-controlled genes by palmitate in synchronized hepatocytes.

**Table 2:** Circadian amplitude of the key clock genes in Hepa1 cells treated with either BSA or Palmitate.

Clock gene	Amplitude-BSA	Amplitude-Palmitate
*Dbp*	1.642425	0.421676
*Per2*	0.103846	0.407251
*Nr1d1*	0.686134	0
*Pgc1α*	0	0.046985
*Cry1*	0.279046	0.563133
*Cry2*	0.116164	0.394407
*Bmal1*	0.126013	0.204212
*Clock*	0.079542	0.039975

### Palmitate does not reduce the BMAL1 and CLOCK protein abundance and alter their nuclear localization

**Fig 3:**
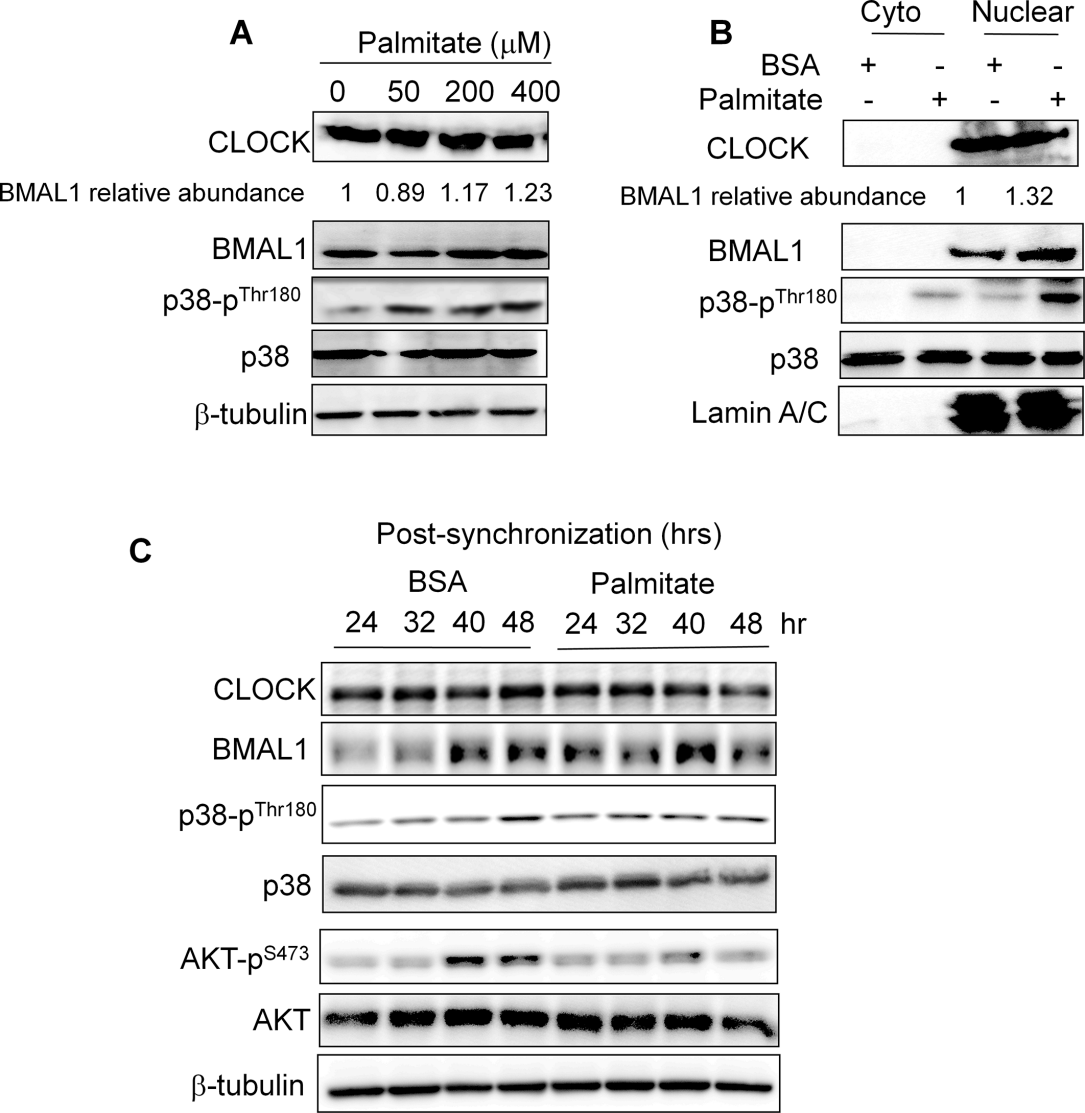
Effects of palmitate treatment on the protein abundance, nuclear localization, and oscillations of BMAL1 and CLOCK in hepatocytes. **(A)** Effects of palmitate on the levels of the endogenous BMAL1 and CLOCK in hepatocytes. Hepa1 cells were treated with an increasing dose of palmitate for 6 hr. Abundance of CLOCK, BMAL1, p38, and p38-P was detected in lysates by immunoblotting. β-tubulin was used as loading control. The relative BMAL1 abundance over loading control was quantified and labeled above the BMAL1 blot. **(B)** Effects of palmitate on both cytoplasmic and nuclear abundance of BMAL1 and CLOCK in hepatocytes. Following palmitate treatment at 200 μM for 6 hrs, cytoplasmic and nuclear fractions from Hepa1 cell lysates were used for detection of CLOCK, BMAL1, p38, and p38-P by immunoblotting. Lamin A/C was detected as a marker for nuclear fraction. **(C)** Effects of palmitate on the circadian oscillations of BMAL1 and CLOCK proteins in synchronized hepatocytes. 2 hr after synchronization by serum shock, Hepa1 cells were treated with BSA or palmitate at 50 μM and then harvested at 24 hr, 32 hr, 40 hr, and 48 hr. The levels of AKT-P, AKT, p38-P, and p38 were examined as well.

Functioning as a transcriptional-translational feedback loop, the molecular clock is resistant to perturbations by adjusting the expression or activity of its components [55]. Since palmitate treatment does not seem to impact the *Bmal1* mRNA expression in PMHs (**Fig 1B**), we suspect that palmitate might alter the BMAL1 and CLOCK protein expression and subsequently suppress their transcriptional function. To our surprise, increasing dosage of palmitate treatment fails to affect the protein level of CLOCK but slightly increases overall levels of BMAL1 protein (about 1.2 fold) **(Fig 3A).** Furthermore, in the presence of palmitate treatment, both of proteins are still predominantly localized inside the nucleus (**Fig 3B**), where these two proteins form a transcriptional complex to activate their targets. In the meanwhile, we detected a robust p38-P in acute palmitate-treated hepatocytes, a positive response to acute palmitate treatment [51]. Next, we tested whether chronic palmitate exposure affects protein oscillations of BMAL1 and CLOCK in synchronized Hepa1 cells. As shown in **Fig 3C**, levels of CLOCK protein remain constant, while BMAL1 protein displays robust oscillations in BSA-treated synchronized Hepa1 cells. However, palmitate treatment renders BMAL1 protein level constant throughout the cycle without showing effects on CLOCK. Meanwhile, chronic palmitate treatment modestly elevates p38 phosphorylation but greatly dampens AKT phosphorylation in those cells, consistent with recent findings that impairment of the hepatic circadian clock reduces AKT phosphorylation [56, 57]. Our results indicate that palmitate represses the hepatic molecular clock without reducing the overall protein abundance of nuclear BMAL1 and CLOCK in hepatocytes. Rather, we observed that palmitate blunts the oscillations of BMAL1 protein in synchronized hepatocytes.

### Palmitate disrupts BMAL1:CLOCK interaction in hepatocytes

**Fig 4:**
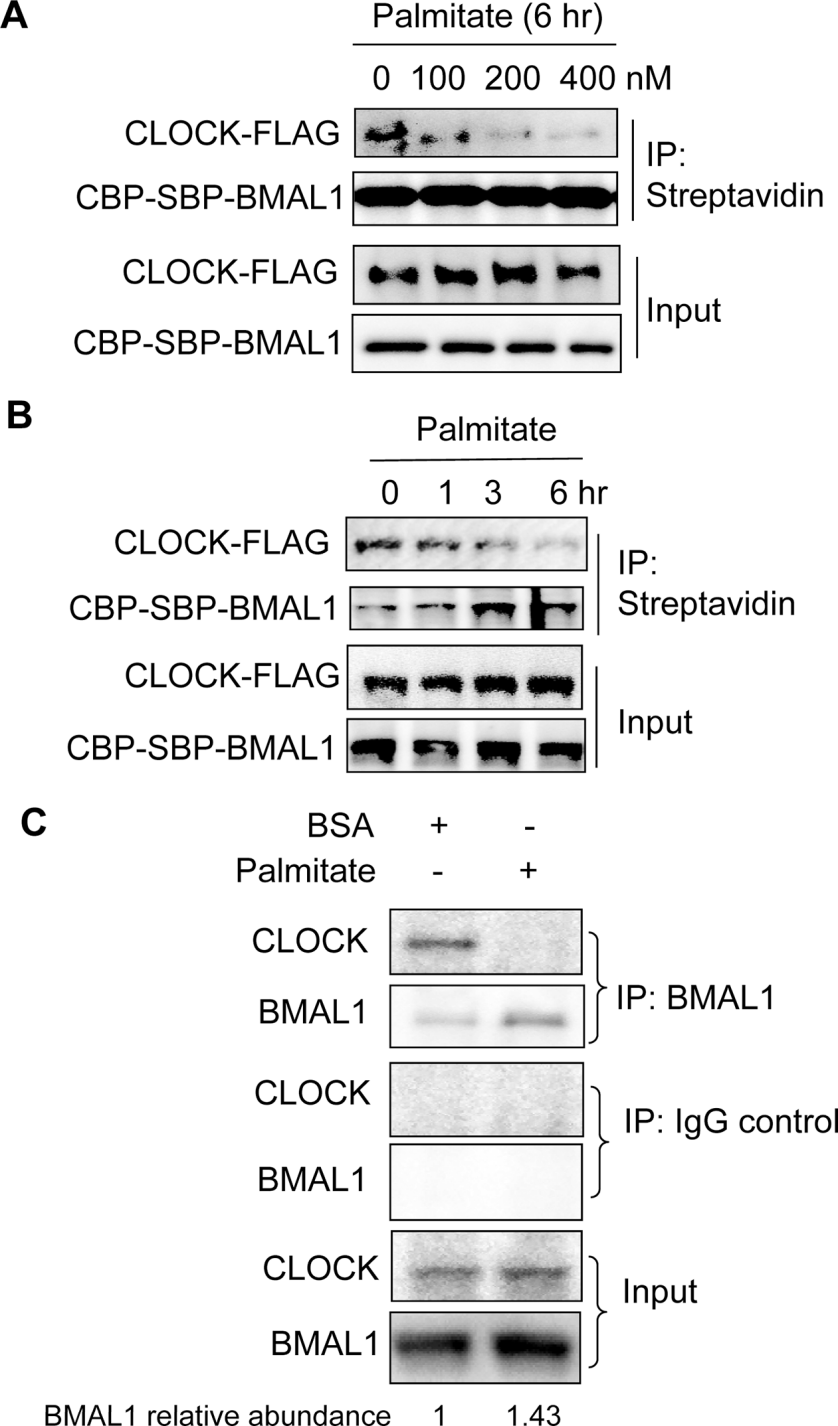
Palmitate disrupts BMAL1-CLOCK protein interaction in hepatocytes. (**A**) Palmitate disrupts BMAL1-CLOCK interaction in a dose-dependent manner in hepatocytes. Hepa1 cells were transfected with CBP-SBP-Bmal1 and Clock-Flag. 36 h later, cells were treated with an increasing dose of palmitate for 6 hr. Cell lysates were subjected to immunoprecipitation with Streptavidin beads and the presence of CLOCK-FLAG and CBP-SBP-BMAL1 was detected by anti-FLAG and anti-CBP respectively. (**B**) Palmitate disrupts BMAL1-CLOCK interaction in a time-dependent manner in hepatocytes. After transfection with CBP-SBP-Bmal1 and Clock-Flag, Hepa1 cells were treated with palmitate for 0 hr, 2 hr, 4hr, and 6 hr and subjected to immunoprecipitation with Streptavidin beads. CBP-SBP-BMAL1 and CLOCK-FLAG were determined as above. (**C**) Palmitate disrupts the endogenous BMAL1-CLOCK protein complex formation in hepatocytes. Hepa1 cells were synchronized first and exposed to BSA or palmitate for 24 hr. Cells were then lysed and subjected to immunoprecipitation with anti-BMAL1 and immunoblotting with anti-CLOCK. The relative BMAL1 expression over loading control was quantified and labeled underneath BMAL1 blot.

The BMAL1:CLOCK protein complex is the major driving force for the oscillations of *Per1* and *Dbp* [34–36]. So far, we have shown that palmitate suppresses BMAL1-CLOCK transcriptional activity without altering the abundance or localization of BMAL1 and CLOCK. This prompted us to examine whether palmitate may influence the BMAL1:CLOCK complex formation in hepatocytes. To this end, we treated Hepa1 cells with palmitate in a dose- and time-dependent manner after co-transfection with CBP (calmodulin-binding protein)- and SBP (streptavidin-binding protein)-tagged Bmal1 and Clock-Flag. We then performed immune-precipitation with streptavidin beads to capture CBP-SBP-tagged BMAL1 and its interacting proteins. As shown in **Fig 4A and 4B**, palmitate treatment as low as 100 μM or as short as 2 hr is sufficient to abolish BMAL1-CLOCK interaction. To confirm this finding at the endogenous level, we performed another immunoprecipitation assay with anti-BMAL1 antibody in Hepa1 cells following 24-hr palmitate treatment. Consistent with over-expression conditions, protein interaction between the endogenous BMAL1 and CLOCK is largely abolished in palmitate-treated Hepa1 cells even though the protein levels for both proteins are comparable in inputs (**Fig 4C**). All these results suggest that the dampened clock gene expression during palmitate treatment may be mediated through a loss of BMAL1:CLOCK interaction and subsequent inactivation of BMAL1:CLOCK-dependent transcription.

### Suppression of SIRT1 mimics the effects of palmitate on BMAL1-CLOCK interaction and activity

**Fig 5:**
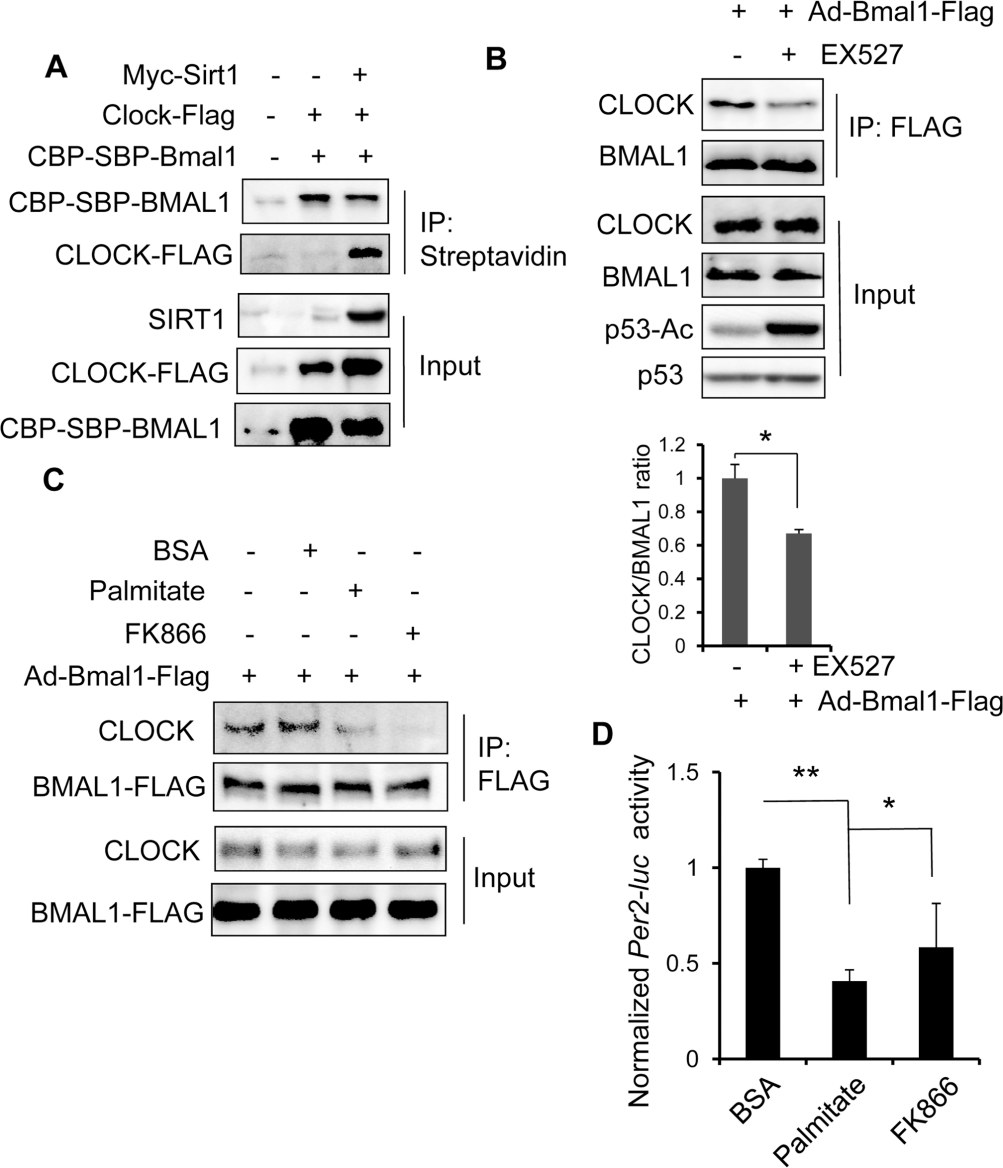
Suppression of SIRT1 activity mimics the palmitate effects on BMAL1-CLOCK interaction and transcriptional activity in hepatocytes. (**A**) BMAL1-CLOCK complex formation requires SIRT1 in 293T cells. 293T cells were co-transfected with CBP-SBP-Bmal1 and Clock-Flag in the presence and absence of Myc-Sirt1. 36 hr later, cells were harvested and subjected to immunoprecipitation with Streptavidin beads to capture CBP-SBP-Bmal1. CBP-SBP-BMAL1, CLOCK-FLAG, Myc-SIRT1 was detected by anti-CBP, anti-FLAG, and anti-Myc immunoblotting. (**B**) SIRT1 inhibition disrupts the formation of the BMAL1:CLOCK complex in hepatocytes. Hepa1 cells were synchronized with serum shock and treated with EX527 (100 nM) or vehicle control for 6 hr after Ad-Bmal1-Flag transduction. Protein lysates were used in immunoprecipitation with anti-FLAG and detection of the endogenous CLOCK with anti-CLOCK. The level of p53-Ac in input was detected to confirm EX527-induced SIRT1 inhibition. The experiment was repeated for three times. The ration of CLOCK over BMAL1 were quantified from 3 independent experiments and shown in the bar graph on the right. *p < 0.05. (**C**) Inhibition of NAD biosynthesis disrupts the BMAL1-CLOCK interaction. PMHs were transduced with Ad-Bmal1-Flag for 24 hr before exposure to BSA, palmitate (200 μM), or FK886 (500 nM) for additional 6 hr. Cells were harvested for immunoprecipitation with Anti-FLAG and the presence of CLOCK was detected by anti-CLOCK. (**D**) Inhibition of NAD biosynthesis impairs activation of *Per2-luc* by BMAL1-CLOCK. Hepa1 cells were transfected with *Per2-luc* along with Bmal1 and Clock expression vectors. 36 h later, cells were treated with either palmitate at 50 μM or FK886 at 500 nM for 12 hr before luciferase assay. Luciferase activity was normalized to β-gal activity. Data were plotted as mean + SD (n = 4). * *p* value < 0.05 and ** *p* value < 0.01.

SIRT1 has been shown to be a regulator of BMAL1: CLOCK transcriptional activity [41, 58]. SIRT1 expression or activity has been shown to be reduced or inhibited in obesity or after palmitate exposure [59–61]. To test whether SIRT1 might be involved in palmitate-induced repression of BMAL1-CLOCK interaction, we overexpressed *Sirt1* in 293T cells in which we detected a very low level of the endogenous SIRT1. As shown in **Fig 5A**, streptavidin beads are unable to pull down CLOCK-FLAG in cells cotransfected with both CBP-SBP-Bmal1 and Clock-Flag. However, significant amount of CLOCK-FLAG is present in immuno-precipitated CBP-SBP-Bmal1 in the presence of Sirt1 overexpression. We next used SIRT1-specific inhibitor EX527 [61–63] to test how inhibition of SIRT1 activity affects BMAL1-CLOCK interaction in PMH hepatocytes transduced with Ad-Bmal1-Flag. While elevated levels of acetylated p53 after SIRT1 suppression by EX527 is consistent with the literature [63], a lower amount of the endogenous CLOCK is shown to interact with FLAG-tagged BMAL1 after immuno-precipitation with anti-FLAG (**Fig 5B**). Next, we used FK866, an NAD biosynthesis inhibitor [64] to further test how blocking SIRT1 affects the clock protein interaction and activity in comparison with palmitate treatment. In PMH cells transduced with Ad-Bmal1-Flag, both palmitate and FK866 are able to disrupt the interaction between the endogenous CLOCK and BMAL1-FLAG (**Fig 5C**). Consistently, treatment with either palmitate (200 μM) or FK866 (500 nM) for 6 hr blocks the *Per2-luc* activation in Hepa1 cells transfected with both Bmal1 and Clock overexpression constructs **(Fig 5D**). Thus, we generated evidence that SIRT1 suppression has similar effects on the circadian clock as palmitate treatment, in support of the notion that SIRT1 might be targeted by palmitate to inhibit the clock function in hepatocytes.

### Palmitate impairs BMAL-CLOCK interaction and function in a SIRT1-dependent manner

**Fig 6:**
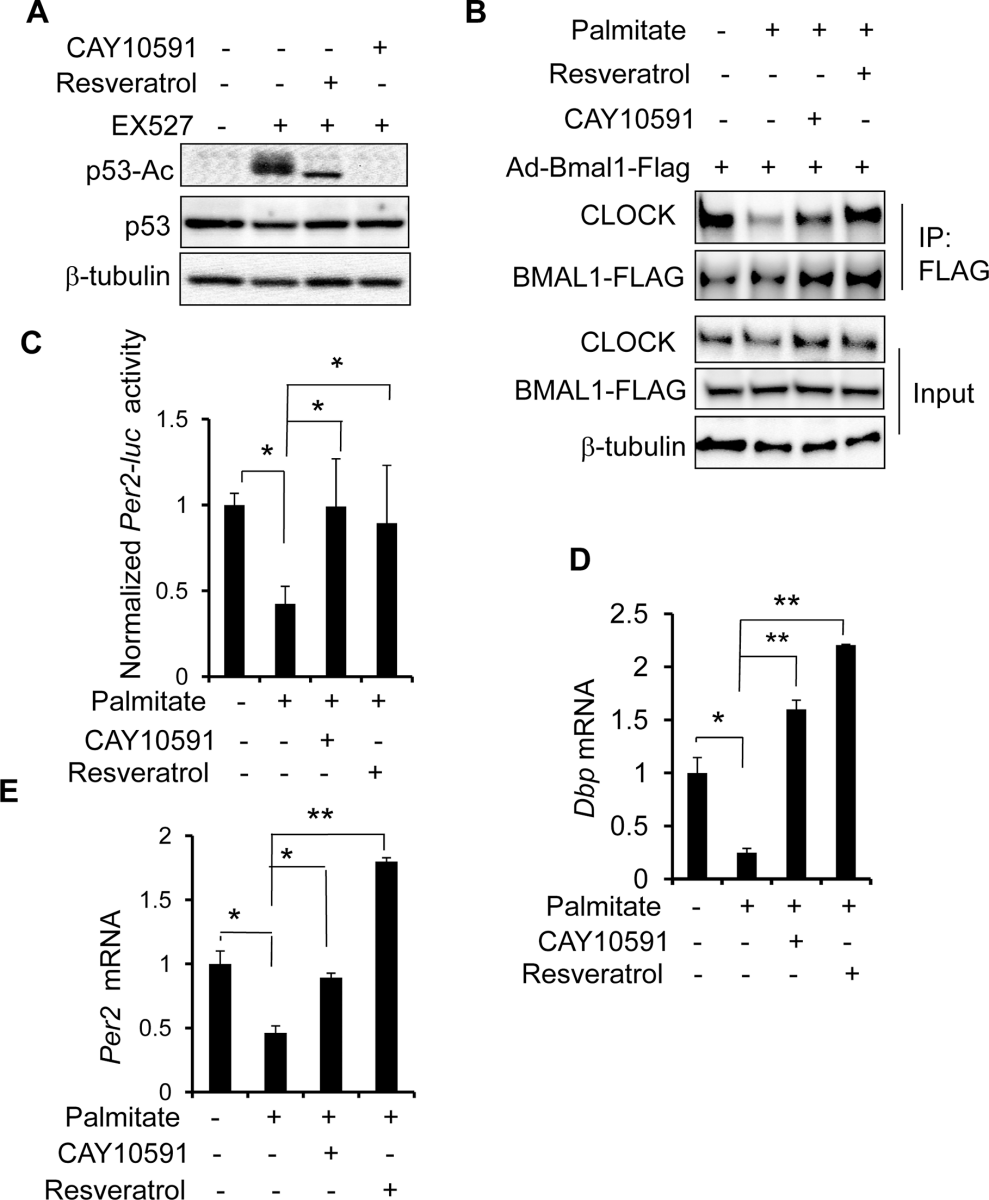
Pharmacological activation of SIRT1 rescues palmitate-induced inhibition of BMAL1-CLOCK interaction and transcriptional activities in hepatocytes. **(A)** Effects of CAY10591 and resverotal on SIRT1 activity. After exposure to EX527 at 100 nM for 3 hr, Hepa1 cells were treated with either CAY10591 at 60 μM or resveratrol at 25 μM for additional 3 hr. Cell lysates were used to examine the levels of total p53 and p53-Ac, a marker for the intracellular SIRT1 activity. **(B)** SIRT1 activators restore BMAL1-CLOCK interaction in palmitate-treated hepatocytes. After transduction with Ad-BMAL1-Flag for 16 hr, PMHs were treated with BSA or palmitate for 16 hr before adding CAY10591 or resveratrol or vehicle for additional 6 hr. Cells were then harvested for co-immunoprecipitation assay with anti-FLAG to detect the BMAL1-CLOCK complex. **(C)** SIRT1 activators reverse the inhibition of *Per2-luc* by palmitate in hepatocytes. After co-transfection with *Per2-luc* and expression vectors of Bmal1 and Clock, Hepa1 were exposed to either BSA or palmitate for 16 hr and then treated with either CAY10591 or resveratrol for another 8 hr. Luciferase activity was normalized to β–gal activity. Data were plotted as mean + SD (n = 3). **(D-E)** SIRT1 activation abrogates palmitate-induced suppression of clock genes. PMH cells were treated with BSA or palmitate for 16 hr prior to addition of CAY10591 or resveratrol for another 8 hr. Cells were harvested for mRNA extraction and gene expression by RT-qPCR. The results were plotted as fold change using the value of BSA-treated samples as 1. Data were plotted as mean + SD (n = 4). *p < 0.05 and ** *p* < 0.01.

So far, we have shown that palmitate attenuates the molecular clock function in hepatocytes likely via disrupting the BMAL1:CLOCK complex formation. We also showed that in hepatocytes SIRT1 plays a critical role in enhancing BMAL1:CLOCK protein-protein interaction and inhibition of SIRT1 mimics the palmitate suppression of BMAL1:CLOCK interaction and function. Conceivably, SIRT1 activation might counteract the effects of palmitate on BMAL1:CLOCK protein-protein interaction. To test this possibility, we used two different SIRT1 activators, CAY10591 [65, 66] and resveratrol [67, 68]. To validate their effects on SIRT1 activation, we measured the levels of acetylated p53 (p53-Ac), a direct intracellular SIRT1 substrate, in hepatocytes. Indeed, Inhibition of SIRT1 by EX527 elevates the level of p53-Ac, whereas co-treatment with either CAY10591 or resveratrol reduces p53-Ac levels to the basal (**Fig 6A**). Specifically, we examined the effects of treatment with either SIRT1 activator on the BMAL1-CLOCK complex during palmitate treatment. In PMH cells transduced with Ad-Bmal1-Flag, palmitate treatment alone consistently reduces BMAL1-FLAG interaction with the endogenous CLOCK protein, whereas either CAY10591 or resveratrol treatment restores interaction of both proteins (**Fig 6B**). We next tested whether both activators could reverse the palmitate effects on the BMAL1:CLOCK-mediated transcriptional activity. Activation of SIRT1 with either CAY10591 or resveratrol following palmitate treatment reverses suppression of the *Per2-luc* activity driven by overexpression of both BMAL1 and CLOCK in Hepa1 (**Fig 6C**). Furthermore, CAY10591 and resveratrol block suppression of the endogenous *Dbp* and *Per2* expression in palmitated-treated Hepa1 cells (**Fig 6D and 6E**). Take all together, our data showed that pharmacological activation of SIRT1 mitigates the negative impact of palmitate on the molecular clock in hepatocytes.

## Discussion

In this study we demonstrated the palmitate-induced inhibition of the molecular circadian clock and identified SIRT1 as a downstream target in hepatocytes. We first showed that palmitate alone is sufficient to interfere with BMAL1-CLOCK interaction and suppress the circadian clock function in hepatocytes. Inhibition of intracellular SIRT1 activity by EX527 or FK866 dissociates the BMAL1-CLOCK complex and suppresses the *Per2-luc* reporter activity, whereas SIRT1-specific activators reverse the palmitate-induced suppression on the circadian clock in hepatocytes. Thus, our work supports that palmitate inhibits the circadian clock function by disrupting SIRT1-dependent BMAL1-CLOCK transcription complex formation and further strengthens the link between the circadian clock and metabolism.

Elevation of serum palmitate level gives rise to lipotoxicity in HFD-fed mice [50], which is characterized as enhanced inflammation, ER stress, ROS generation, and cell death [2, 3, 7, 11, 53]. Lipotoxicity has been proposed to promote the development of insulin resistance in the liver and skeletal muscles. Palmitate-induced-lipotoxicity varies among cell lines in a dose- and time-dependent fashion. No study has been reported to examine the effects of palmitate on the circadian clock in hepatocytes. In our *in vitro* study with primary mouse hepatocytes and cultured hepatoma cell lines, we observed that palmitate is able to potently impair the molecular clock function. Such observation is concordant with a previous report showing that palmitate suppresses clock gene expression in cultured hypothalamic neurons [12, 13], highlighting that palmitate represses clock genes in multiple cell types although the underlying mechanism might be different. In the case of hepatocytes, our results support that palmitate may suppress clock gene oscillations by disrupting the BMAL1:CLOCK complex formation. Since elevated level of serum palmitate was often found in obese and insulin-resistant animal models, it would be of great interest to examine hepatic BMAL1-CLOCK interactions in liver tissues of those animals.

The second important observation from our study is that palmitate treatment disrupts the BMAL1-CLOCK complex formation in a SIRT1-dependent manner. As bHLH transcription activators, BMAL1 and CLOCK heterodimerize via the PAS domains in the cytosol and translocate into the nucleus [36]. Direct BMAL1-CLOCK interaction has been demonstrated in vitro and by analysis of protein structure [69]. Our study indicates that SIRT1 activity is required for maintaining a stable BMAL1-CLOCK interaction in hepatocytes. SIRT1 has been shown to remove the acetyl group from both histone and non-histone targets [43]. We suspect that enhanced acetylation of either BMAL1 or CLOCK or both may hinder their interaction or destabilize the newly formed BMAL1-CLOCK complex in palmitate-treated hepatocytes. In a previous study, SIRT1 was found to deacetylate the C-terminal of BMAL1 at lysine 537 that is the target for the intrinsic HAT activity of CLOCK [70]. However, the C-terminal of BMAL1 protein is not absolutely required for its complex formation with CLOCK protein [69, 71]. As a result, we suspect that lysine residues within either the bHLH or PAS domains may also be de-acetylated by SIRT1 to maintain the stable complex of BMAL1-CLOCK. To that end, we will conduct mass spectrometry analysis to assess the acetylation status of BMAL1 and CLOCK proteins before and after palmitate treatment in hepatocytes. This unbiased approach may be able to identify the specific lysine residues within interaction domains of either BMAL1 or CLOCK protein that are targeted by SIRT1.

So far, a reciprocal regulation has been proposed between the NAD-SIRT1 pathway and the circadian clock. On one hand, BMAL1-CLOCK controls circadian oscillations of NAMPT, the salvage pathway, and intracellular NAD [58, 72]. On the other hand, SIRT1 has been shown to directly promote deacetylation of BMAL1 and PER2 to regulate BMAL1 binding and PER2 protein degradation [40, 41, 58]. Our data revealed that SIRT1 inhibition by EX527 or FK866 reduces BMAL1-CLOCK interaction in hepatocytes, whereas SIRT1 activator restores BMAL1-CLOCK interaction in palmitate-treated hepatocytes. Thus, our work identified another layer of circadian regulation that SIRT1 activity is crucial for maintaining the stable complex of BMAL1-CLOCK in hepatocytes. Since SIRT1 is sensitive to nutritional status and cellular stress, modulation of its activity might be a sensitive way to fine-tune the molecular clock in hepatocytes in response to environmental cues. Of note, SIRT1 activation is not always associated with increased oscillation of circadian genes [41, 58, 72]. In MEFs, pharmacological activation of SIRT1 in fact actually reduces oscillations of *Dbp* [42]. Another study showed that acutely knockdown of *Sirt1* expression in neuro-blastoma N2a cells greatly dampens oscillations of *Per2* and *Nr1d1* [73], consistent with our data in hepatocytes. Why MEFs behave differently from neuronal cells or hepatocytes in terms of the effects of SIRT1 activation on clock genes is currently unknown. We suspect that it is likely due to the differences in either basal levels of SIRT1 activity or SIRT1 interaction networks in different cell types [44]. Further investigation on the cell-type specific responses to SIRT1 modulators regarding the clock gene expression is needed.

Currently, whether and how palmitate inhibits SIRT1 activity remains unclear. It has been reported that JNK activation inhibits SIRT1 function through direct phosphorylation [59]. It has also been suggested that palmitate induces oxidative stress to inactivate SIRT1 via cysteine modification [60]. Additionally, palmitoylation has been shown to regulate protein localization, interaction, and function [74]. It is tantalizing to speculate that exposure to palmitate could also lead to inhibition of SIRT1 activity through palmitoylation. We will investigate how palmitate affects post-translational modifications of SIRT1 protein in the future.

Our work for the first time revealed that SIRT1 activity is critical for maintaining the stable BMAL1-CLOCK complex during nutritional stress in hepatocytes. Our study demonstrated that SIRT1 activators allay the palmitate-induced repression of the molecular clock in hepatocytes. It should be noted that in vitro cultured hepatocytes may differ from hepatocytes in vivo regarding their sensitivity to palmitate-induced SIRT1 inhibition. At the meantime, hepatocytes in vivo may also respond differently towards chemical SIRT1 modulators in terms of dose and duration. Also, we are keenly aware of the limitations of using pharmacological reagents to manipulate SIRT1 activity in hepatocytes. In spite of these limitations, our current work provides a potential molecular explanation for the detrimental effect of chronic high-fat diet feeding on the hepatic circadian cock, In the future, we will use mouse models with genetically altered expression of *Sirt1* in the liver to determine whether activation of SIRT1 could be a valid approach to restore the hepatic clock function in response to high-fat diet in vivo.

## References

[1] Samuel VT, Shulman GI. Mechanisms for insulin resistance: common threads and missing links. Cell. 2012;148(5):852–71. Epub 2012/03/06. doi: S0092-8674(12)00217-6 [pii] 10.1016/j.cell.2012.02.017 22385956 PMC3294420

[2] Savage DB, Petersen KF, Shulman GI. Disordered lipid metabolism and the pathogenesis of insulin resistance. Physiol Rev. 2007;87(2):507–20. Epub 2007/04/13. doi: 87/2/507 [pii] 10.1152/physrev.00024.2006 17429039 PMC2995548

[3] Samuel VT, Petersen KF, Shulman GI. Lipid-induced insulin resistance: unravelling the mechanism. Lancet. 375(9733):2267–77. Epub 2010/07/09. doi: S0140-6736(10)60408-4 [pii] 10.1016/S0140-6736(10)60408-4 20609972 PMC2995547

[4] Kraegen EW, Cooney GJ, Ye J, Thompson AL. Triglycerides, fatty acids and insulin resistance—hyperinsulinemia. Exp Clin Endocrinol Diabetes. 2001;109(4):S516–26. Epub 2001/07/17 . 11453039 10.1055/s-2001-15114

[5] Kraegen EW, Cooney GJ, Ye JM, Thompson AL, Furler SM. The role of lipids in the pathogenesis of muscle insulin resistance and beta cell failure in type II diabetes and obesity. Exp Clin Endocrinol Diabetes. 2001;109 Suppl 2:S189–201. Epub 2001/07/20. 10.1055/s-2001-18581 . 11460570

[6] Hirosumi J, Tuncman G, Chang L, Gorgun CZ, Uysal KT, Maeda K, et al A central role for JNK in obesity and insulin resistance. Nature. 2002;420(6913):333–6. Epub 2002/11/26. doi: 10.1038/nature01137 nature01137 [pii] . 12447443 10.1038/nature01137

[7] Boden G, She P, Mozzoli M, Cheung P, Gumireddy K, Reddy P, et al Free fatty acids produce insulin resistance and activate the proinflammatory nuclear factor-kappaB pathway in rat liver. Diabetes. 2005;54(12):3458–65. Epub 2005/11/25. doi: 54/12/3458 [pii] . 16306362 10.2337/diabetes.54.12.3458

[8] Ozcan U, Cao Q, Yilmaz E, Lee AH, Iwakoshi NN, Ozdelen E, et al Endoplasmic reticulum stress links obesity, insulin action, and type 2 diabetes. Science. 2004;306(5695):457–61. Epub 2004/10/16. doi: 306/5695/457 [pii] 10.1126/science.1103160 . 15486293

[9] Kim JK, Fillmore JJ, Chen Y, Yu C, Moore IK, Pypaert M, et al Tissue-specific overexpression of lipoprotein lipase causes tissue-specific insulin resistance. Proc Natl Acad Sci U S A. 2001;98(13):7522–7. Epub 2001/06/08. doi: 10.1073/pnas.121164498 121164498 [pii] 11390966 10.1073/pnas.121164498 PMC34701

[10] Turinsky J, Bayly BP, O'Sullivan DM. 1,2-Diacylglycerol and ceramide levels in rat skeletal muscle and liver in vivo. Studies with insulin, exercise, muscle denervation, and vasopressin. J Biol Chem. 1990;265(14):7933–8. Epub 1990/05/15 . 2186032

[11] Nakamura S, Takamura T, Matsuzawa-Nagata N, Takayama H, Misu H, Noda H, et al Palmitate induces insulin resistance in H4IIEC3 hepatocytes through reactive oxygen species produced by mitochondria. J Biol Chem. 2009;284(22):14809–18. Epub 2009/04/01. doi: M901488200 [pii] 10.1074/jbc.M901488200 19332540 PMC2685662

[12] Fick LJ, Fick GH, Belsham DD. Palmitate alters the rhythmic expression of molecular clock genes and orexigenic neuropeptide Y mRNA levels within immortalized, hypothalamic neurons. Biochem Biophys Res Commun. 413(3):414–9. Epub 2011/09/07. doi: S0006-291X(11)01512-9 [pii] 10.1016/j.bbrc.2011.08.103 . 21893042

[13] Greco JA, Oosterman JE, Belsham DD. Differential effects of omega-3 fatty acid docosahexaenoic acid and palmitate on the circadian transcriptional profile of clock genes in immortalized hypothalamic neurons. American journal of physiology Regulatory, integrative and comparative physiology. 2014;307(8):R1049–60. 10.1152/ajpregu.00100.2014 25144192 PMC4200380

[14] Challet E. Circadian clocks, food intake, and metabolism. Prog Mol Biol Transl Sci. 2013;119:105–35. Epub 2013/08/01. doi: B978-0-12-396971-2.00005–1 [pii] 10.1016/B978-0-12-396971-2.00005-1 . 23899596

[15] Dibner C, Schibler U, Albrecht U. The mammalian circadian timing system: organization and coordination of central and peripheral clocks. Annu Rev Physiol. 2010;72:517–49. Epub 2010/02/13. 10.1146/annurev-physiol-021909-135821 . 20148687

[16] Lamia KA, Storch KF, Weitz CJ. Physiological significance of a peripheral tissue circadian clock. Proc Natl Acad Sci U S A. 2008;105(39):15172–7. Epub 2008/09/10. doi: 0806717105 [pii] 10.1073/pnas.0806717105 18779586 PMC2532700

[17] Marcheva B, Ramsey KM, Buhr ED, Kobayashi Y, Su H, Ko CH, et al Disruption of the clock components CLOCK and BMAL1 leads to hypoinsulinaemia and diabetes. Nature. 466(7306):627–31. Epub 2010/06/22. doi: nature09253 [pii] 10.1038/nature09253 20562852 PMC2920067

[18] Rudic RD, McNamara P, Curtis AM, Boston RC, Panda S, Hogenesch JB, et al BMAL1 and CLOCK, two essential components of the circadian clock, are involved in glucose homeostasis. PLoS Biol. 2004;2(11):e377 Epub 2004/11/04. 10.1371/journal.pbio.0020377 15523558 PMC524471

[19] Shimba S, Ogawa T, Hitosugi S, Ichihashi Y, Nakadaira Y, Kobayashi M, et al Deficient of a clock gene, brain and muscle Arnt-like protein-1 (BMAL1), induces dyslipidemia and ectopic fat formation. PLoS One. 6(9):e25231 Epub 2011/10/04. doi: 10.1371/journal.pone.0025231 PONE-D-11-09950 [pii] 21966465 10.1371/journal.pone.0025231 PMC3178629

[20] Turek FW, Joshu C, Kohsaka A, Lin E, Ivanova G, McDearmon E, et al Obesity and metabolic syndrome in circadian Clock mutant mice. Science. 2005;308(5724):1043–5. Epub 2005/04/23. doi: 1108750 [pii] 10.1126/science.1108750 . 15845877 PMC3764501

[21] Grimaldi B, Bellet MM, Katada S, Astarita G, Hirayama J, Amin RH, et al PER2 controls lipid metabolism by direct regulation of PPARgamma. Cell Metab. 12(5):509–20. Epub 2010/11/03. doi: S1550-4131(10)00357-8 [pii] 10.1016/j.cmet.2010.10.005 . 21035761 PMC4103168

[22] Kim EJ, Yoon YS, Hong S, Son HY, Na TY, Lee MH, et al. RORalpha-induced activation of AMP-activated protein kinase results in attenuation of hepatic steatosis. Hepatology. Epub 2011/12/21. 10.1002/hep.25529 . 22183856

[23] Lau P, Fitzsimmons RL, Raichur S, Wang SC, Lechtken A, Muscat GE. The orphan nuclear receptor, RORalpha, regulates gene expression that controls lipid metabolism: staggerer (SG/SG) mice are resistant to diet-induced obesity. J Biol Chem. 2008;283(26):18411–21. Epub 2008/04/29. doi: M710526200 [pii] 10.1074/jbc.M710526200 . 18441015

[24] Feng D, Liu T, Sun Z, Bugge A, Mullican SE, Alenghat T, et al A circadian rhythm orchestrated by histone deacetylase 3 controls hepatic lipid metabolism. Science. 331(6022):1315–9. Epub 2011/03/12. doi: 331/6022/1315 [pii] 10.1126/science.1198125 21393543 PMC3389392

[25] Le Martelot G, Claudel T, Gatfield D, Schaad O, Kornmann B, Sasso GL, et al REV-ERBalpha participates in circadian SREBP signaling and bile acid homeostasis. PLoS Biol. 2009;7(9):e1000181 Epub 2009/09/02. 10.1371/journal.pbio.1000181 19721697 PMC2726950

[26] Froy O. The relationship between nutrition and circadian rhythms in mammals. Front Neuroendocrinol. 2007;28(2–3):61–71. Epub 2007/04/25. doi: S0091-3022(07)00004-0 [pii] 10.1016/j.yfrne.2007.03.001 . 17451793

[27] Huang W, Ramsey KM, Marcheva B, Bass J. Circadian rhythms, sleep, and metabolism. J Clin Invest. 121(6):2133–41. Epub 2011/06/03. doi: 46043 [pii] 10.1172/JCI46043 21633182 PMC3104765

[28] Challet E. Interactions between light, mealtime and calorie restriction to control daily timing in mammals. J Comp Physiol B. 180(5):631–44. Epub 2010/02/23. 10.1007/s00360-010-0451-4 . 20174808

[29] Mendoza J. Circadian clocks: setting time by food. J Neuroendocrinol. 2007;19(2):127–37. Epub 2007/01/12. doi: JNE1510 [pii] 10.1111/j.1365-2826.2006.01510.x . 17214875

[30] Hsieh MC, Yang SC, Tseng HL, Hwang LL, Chen CT, Shieh KR. Abnormal expressions of circadian-clock and circadian clock-controlled genes in the livers and kidneys of long-term, high-fat-diet-treated mice. Int J Obes (Lond). 34(2):227–39. Epub 2009/11/11. doi: ijo2009228 [pii] 10.1038/ijo.2009.228 . 19901953

[31] Kohsaka A, Laposky AD, Ramsey KM, Estrada C, Joshu C, Kobayashi Y, et al High-fat diet disrupts behavioral and molecular circadian rhythms in mice. Cell Metab. 2007;6(5):414–21. Epub 2007/11/07. doi: S1550-4131(07)00266-5 [pii] 10.1016/j.cmet.2007.09.006 . 17983587

[32] Yanagihara H, Ando H, Hayashi Y, Obi Y, Fujimura A. High-fat feeding exerts minimal effects on rhythmic mRNA expression of clock genes in mouse peripheral tissues. Chronobiol Int. 2006;23(5):905–14. Epub 2006/10/20. doi: M6X0146501105014 [pii] 10.1080/07420520600827103 . 17050208

[33] Eckel-Mahan KL, Patel VR, de Mateo S, Orozco-Solis R, Ceglia NJ, Sahar S, et al Reprogramming of the circadian clock by nutritional challenge. Cell. 2013;155(7):1464–78. 10.1016/j.cell.2013.11.034 . 24360271 PMC4573395

[34] Dibner C, Schibler U, Albrecht U. The mammalian circadian timing system: organization and coordination of central and peripheral clocks. Annu Rev Physiol. 72:517–49. Epub 2010/02/13. 10.1146/annurev-physiol-021909-135821 . 20148687

[35] Reppert SM, Weaver DR. Molecular analysis of mammalian circadian rhythms. Annu Rev Physiol. 2001;63:647–76. Epub 2001/02/22. doi: 10.1146/annurev.physiol.63.1.647 63/1/647 [pii] . 11181971 10.1146/annurev.physiol.63.1.647

[36] Takahashi JS, Hong HK, Ko CH, McDearmon EL. The genetics of mammalian circadian order and disorder: implications for physiology and disease. Nat Rev Genet. 2008;9(10):764–75. Epub 2008/09/20. doi: nrg2430 [pii] 10.1038/nrg2430 . 18802415 PMC3758473

[37] Li S, Lin JD. Molecular control of circadian metabolic rhythms. J Appl Physiol. 2009;107(6):1959–64. Epub 2009/07/04. doi: 00467.2009 [pii] 10.1152/japplphysiol.00467.2009 . 19574505

[38] Maury E, Ramsey KM, Bass J. Circadian rhythms and metabolic syndrome: from experimental genetics to human disease. Circulation research. 2010;106(3):447–62. 10.1161/CIRCRESAHA.109.208355 20167942 PMC2837358

[39] Sahar S, Sassone-Corsi P. Metabolism and cancer: the circadian clock connection. Nature reviews Cancer. 2009;9(12):886–96. 10.1038/nrc2747 . 19935677

[40] Asher G, Gatfield D, Stratmann M, Reinke H, Dibner C, Kreppel F, et al SIRT1 regulates circadian clock gene expression through PER2 deacetylation. Cell. 2008;134(2):317–28. 10.1016/j.cell.2008.06.050 . 18662546

[41] Nakahata Y, Kaluzova M, Grimaldi B, Sahar S, Hirayama J, Chen D, et al The NAD+-dependent deacetylase SIRT1 modulates CLOCK-mediated chromatin remodeling and circadian control. Cell. 2008;134(2):329–40. 10.1016/j.cell.2008.07.002 18662547 PMC3526943

[42] Bellet MM, Nakahata Y, Boudjelal M, Watts E, Mossakowska DE, Edwards KA, et al Pharmacological modulation of circadian rhythms by synthetic activators of the deacetylase SIRT1. Proc Natl Acad Sci U S A. 2013;110(9):3333–8. 10.1073/pnas.1214266110 23341587 PMC3587185

[43] Li X. SIRT1 and energy metabolism. Acta biochimica et biophysica Sinica. 2013;45(1):51–60. 10.1093/abbs/gms108 23257294 PMC3527007

[44] Chen D, Bruno J, Easlon E, Lin SJ, Cheng HL, Alt FW, et al Tissue-specific regulation of SIRT1 by calorie restriction. Genes & development. 2008;22(13):1753–7. 10.1101/gad.1650608 18550784 PMC2492662

[45] Tong X, Muchnik M, Chen Z, Patel M, Wu N, Joshi S, et al Transcriptional repressor E4-binding protein 4 (E4BP4) regulates metabolic hormone fibroblast growth factor 21 (FGF21) during circadian cycles and feeding. J Biol Chem. 2010;285(47):36401–9. Epub 2010/09/21. doi: M110.172866 [pii] 10.1074/jbc.M110.172866 20851878 PMC2978569

[46] Yin L, Joshi S, Wu N, Tong X, Lazar MA. E3 ligases Arf-bp1 and Pam mediate lithium-stimulated degradation of the circadian heme receptor Rev-erb alpha. Proc Natl Acad Sci U S A. 107(25):11614–9. Epub 2010/06/11. doi: 1000438107 [pii] 10.1073/pnas.1000438107 20534529 PMC2895127

[47] Hughes ME, Hogenesch JB, Kornacker K. JTK_CYCLE: an efficient nonparametric algorithm for detecting rhythmic components in genome-scale data sets. J Biol Rhythms. 2010;25(5):372–80. 10.1177/0748730410379711 20876817 PMC3119870

[48] Hatori M, Vollmers C, Zarrinpar A, DiTacchio L, Bushong EA, Gill S, et al Time-restricted feeding without reducing caloric intake prevents metabolic diseases in mice fed a high-fat diet. Cell Metab. 2012;15(6):848–60. Epub 2012/05/23. doi: S1550-4131(12)00189-1 [pii] 10.1016/j.cmet.2012.04.019 22608008 PMC3491655

[49] Hsieh MC, Yang SC, Tseng HL, Hwang LL, Chen CT, Shieh KR. Abnormal expressions of circadian-clock and circadian clock-controlled genes in the livers and kidneys of long-term, high-fat-diet-treated mice. Int J Obes (Lond). 2010;34(2):227–39. Epub 2009/11/11. doi: ijo2009228 [pii] 10.1038/ijo.2009.228 . 19901953

[50] Boden G. Role of fatty acids in the pathogenesis of insulin resistance and NIDDM. Diabetes. 1997;46(1):3–10. Epub 1997/01/01 . 8971073

[51] Gao D, Nong S, Huang X, Lu Y, Zhao H, Lin Y, et al The effects of palmitate on hepatic insulin resistance are mediated by NADPH Oxidase 3-derived reactive oxygen species through JNK and p38MAPK pathways. J Biol Chem. 2010;285(39):29965–73. 10.1074/jbc.M110.128694 20647313 PMC2943261

[52] Yuan H, Zhang X, Huang X, Lu Y, Tang W, Man Y, et al NADPH oxidase 2-derived reactive oxygen species mediate FFAs-induced dysfunction and apoptosis of beta-cells via JNK, p38 MAPK and p53 pathways. PLoS One. 2010;5(12):e15726 Epub 2011/01/07. 10.1371/journal.pone.0015726 21209957 PMC3012098

[53] Cazanave SC, Mott JL, Bronk SF, Werneburg NW, Fingas CD, Meng XW, et al Death receptor 5 signaling promotes hepatocyte lipoapoptosis. J Biol Chem. 2011;286(45):39336–48. Epub 2011/09/24. doi: M111.280420 [pii] 10.1074/jbc.M111.280420 21941003 PMC3234758

[54] Liu C, Li S, Liu T, Borjigin J, Lin JD. Transcriptional coactivator PGC-1alpha integrates the mammalian clock and energy metabolism. Nature. 2007;447(7143):477–81. Epub 2007/05/04. doi: nature05767 [pii] 10.1038/nature05767 . 17476214

[55] Dibner C, Sage D, Unser M, Bauer C, d'Eysmond T, Naef F, et al Circadian gene expression is resilient to large fluctuations in overall transcription rates. EMBO J. 2009;28(2):123–34. Epub 2008/12/17. doi: emboj2008262 [pii] 10.1038/emboj.2008.262 19078963 PMC2634731

[56] Zhang D, Tong X, Arthurs B, Guha A, Rui L, Kamath A, et al Liver clock protein BMAL1 promotes de novo lipogenesis through insulin-mTORC2-AKT signaling. J Biol Chem. 2014;289(37):25925–35. 10.1074/jbc.M114.567628 25063808 PMC4162191

[57] Zhou B, Zhang Y, Zhang F, Xia Y, Liu J, Huang R, et al CLOCK/BMAL1 regulates circadian change of mouse hepatic insulin sensitivity by SIRT1. Hepatology. 2014;59(6):2196–206. 10.1002/hep.26992 . 24442997

[58] Nakahata Y, Sahar S, Astarita G, Kaluzova M, Sassone-Corsi P. Circadian control of the NAD+ salvage pathway by CLOCK-SIRT1. Science. 2009;324(5927):654–7. 10.1126/science.1170803 . 19286518 PMC6501775

[59] Gao Z, Zhang J, Kheterpal I, Kennedy N, Davis RJ, Ye J. Sirtuin 1 (SIRT1) protein degradation in response to persistent c-Jun N-terminal kinase 1 (JNK1) activation contributes to hepatic steatosis in obesity. J Biol Chem. 2011;286(25):22227–34. 10.1074/jbc.M111.228874 21540183 PMC3121368

[60] Shao D, Fry JL, Han J, Hou X, Pimentel DR, Matsui R, et al A redox-resistant sirtuin-1 mutant protects against hepatic metabolic and oxidant stress. J Biol Chem. 2014;289(11):7293–306. 10.1074/jbc.M113.520403 24451382 PMC3953247

[61] Wu L, Zhou L, Lu Y, Zhang J, Jian F, Liu Y, et al Activation of SIRT1 protects pancreatic beta-cells against palmitate-induced dysfunction. Biochim Biophys Acta. 2012;1822(11):1815–25. 10.1016/j.bbadis.2012.08.009 . 22968147

[62] Nie Y, Erion DM, Yuan Z, Dietrich M, Shulman GI, Horvath TL, et al STAT3 inhibition of gluconeogenesis is downregulated by SirT1. Nature cell biology. 2009;11(4):492–500. 10.1038/ncb1857 19295512 PMC2790597

[63] Solomon JM, Pasupuleti R, Xu L, McDonagh T, Curtis R, DiStefano PS, et al Inhibition of SIRT1 catalytic activity increases p53 acetylation but does not alter cell survival following DNA damage. Mol Cell Biol. 2006;26(1):28–38. 10.1128/MCB.26.1.28-38.2006 16354677 PMC1317617

[64] Hasmann M, Schemainda I. FK866, a highly specific noncompetitive inhibitor of nicotinamide phosphoribosyltransferase, represents a novel mechanism for induction of tumor cell apoptosis. Cancer research. 2003;63(21):7436–42 . 14612543

[65] Caruso R, Marafini I, Franze E, Stolfi C, Zorzi F, Monteleone I, et al Defective expression of SIRT1 contributes to sustain inflammatory pathways in the gut. Mucosal immunology. 2014;7(6):1467–79. 10.1038/mi.2014.35 . 24850427

[66] Thompson KJ, Humphries JR, Niemeyer DJ, Sindram D, McKillop IH. The Effect of Alcohol on Sirt1 Expression and Function in Animal and Human Models of Hepatocellular Carcinoma (HCC). Advances in experimental medicine and biology. 2015;815:361–73. 10.1007/978-3-319-09614-8_21 . 25427918

[67] Price NL, Gomes AP, Ling AJ, Duarte FV, Martin-Montalvo A, North BJ, et al SIRT1 is required for AMPK activation and the beneficial effects of resveratrol on mitochondrial function. Cell Metab. 2012;15(5):675–90. 10.1016/j.cmet.2012.04.003 22560220 PMC3545644

[68] Vetterli L, Maechler P. Resveratrol-activated SIRT1 in liver and pancreatic beta-cells: a Janus head looking to the same direction of metabolic homeostasis. Aging. 2011;3(4):444–9 21483037 10.18632/aging.100304 PMC3117460

[69] Huang N, Chelliah Y, Shan Y, Taylor CA, Yoo SH, Partch C, et al Crystal structure of the heterodimeric CLOCK:BMAL1 transcriptional activator complex. Science. 2012;337(6091):189–94. 10.1126/science.1222804 22653727 PMC3694778

[70] Hirayama J, Sahar S, Grimaldi B, Tamaru T, Takamatsu K, Nakahata Y, et al CLOCK-mediated acetylation of BMAL1 controls circadian function. Nature. 2007;450(7172):1086–90. Epub 2007/12/14. doi: nature06394 [pii] 10.1038/nature06394 . 18075593

[71] Dardente H, Fortier EE, Martineau V, Cermakian N. Cryptochromes impair phosphorylation of transcriptional activators in the clock: a general mechanism for circadian repression. The Biochemical journal. 2007;402(3):525–36. 10.1042/BJ20060827 17115977 PMC1863574

[72] Ramsey KM, Yoshino J, Brace CS, Abrassart D, Kobayashi Y, Marcheva B, et al Circadian clock feedback cycle through NAMPT-mediated NAD+ biosynthesis. Science. 2009;324(5927):651–4. 10.1126/science.1171641 19299583 PMC2738420

[73] Chang HC, Guarente L. SIRT1 mediates central circadian control in the SCN by a mechanism that decays with aging. Cell. 2013;153(7):1448–60. 10.1016/j.cell.2013.05.027 23791176 PMC3748806

[74] Chavda B, Arnott JA, Planey SL. Targeting protein palmitoylation: selective inhibitors and implications in disease. Expert opinion on drug discovery. 2014;9(9):1005–19. 10.1517/17460441.2014.933802 . 24967607

